# Distinct contributions of human posterior parietal and dorsal premotor cortex to reach trajectory planning

**DOI:** 10.1038/s41598-019-39188-0

**Published:** 2019-02-13

**Authors:** Artur Pilacinski, Axel Lindner

**Affiliations:** 1 Hertie-Institute for Clinical Brain Research, Department of Cognitive Neurology, Hoppe-Seyler-Str. 3, 72076 Tuebingen, Germany; 2 University Hospital Tuebingen, Department Psychiatry and Psychotherapy, Calwerstraße 14, 72076 Tuebingen, Germany

## Abstract

Goal-directed hand movements are usually directed straight at the target, e.g. when swatting a fly. Their paths can also become quite complex, when drawing or avoiding obstacles. Studies on movement planning have largely neglected the latter movement type and the question of whether it is the same neural machinery that is planning such complex hand trajectories as well as straight, vector-like movements. Using time-resolved fMRI during delayed response tasks we examined planning activity in human superior parietal lobule (SPL) and dorsal premotor cortex (PMd). We show that the recruitment of both areas in trajectory planning differs significantly: PMd represented both straight and complex hand trajectories while SPL only those that led straight to the target. This suggests that while posterior parietal cortex only provides representations for simple, straight reaches, the complex and computationally demanding reach planning necessarily involves dorsal premotor cortex. Our findings yield new insights into the organization of cerebro-cortical strategies of forming reach trajectory plans.

## Introduction

Goal-directed eye saccades and hand reaches share many commonalities. Both movement types are prepared based on target and effector representations in a visual (retinal) reference frame and even the neural correlates responsible for their programming do partially overlap^1^. According to a well-established view, the motor plans for saccades are thereby defined by coding a difference vector between the current position of the eye and the desired saccade endpoint^2–5^. As there are no objects in the eye socket that would interfere with the rotation of the eyeball, such simple planning seems optimal for its purpose. In many cases hand movements are executed in a similar point-to-point fashion, such as when catching a ball or swatting a fly. In these latter situations the hand movement could likewise be determined by a difference vector between target and hand^6^. Yet, such method would not always suffice: imagine you’d like to reach for your pen, but a mug of coffee sits right between the pen and the reaching hand. In such situation your eye could still saccade straight towards the pen while any hand movement aimed just at the endpoint (the pen) would cause your hand to bump into the mug, with potentially severe consequences. Therefore, to allow the hand to circumvent the obstacle, an appropriate reach trajectory needs to be programmed. It seems likely that such ability to precisely plan hand trajectories is not only required to avoid obstacles, but also underlies our ability to perform the endless variety of highly-complex and skillful movements such as drawing or handwriting.

Electrophysiological research in monkeys has yielded some important clues about where and how the planning of reach trajectories could be realized by the brain. A prominent candidate for reach trajectory planning is dorsal premotor cortex (PMd), as neurons in this brain area do represent information relevant for trajectory coding. For instance, in the presence of obstacles PMd does not only code movement plans towards the target location itself but it also represents the initial direction of movement that is needed to circumvent any obstacle^7^. Moreover, Hocherman and Wise^8^ have demonstrated that some neurons in macaque premotor cortex (as well as primary motor cortex and supplementary motor area) exhibit firing patterns that correlate with the curvature of the trajectory of an upcoming reach. Premotor coding of reach curvature may – along with the coding of initial movement direction - support the ability to circumvent obstacles. In accordance with this interpretation, ablation of premotor cortex disables monkeys’ ability to avoid obstacles and they instead attempt to reach directly towards the target^9^. This latter experiment not only directly supports a role of PMd in trajectory planning. It also highlights that planning of straight, direct reaches is still preserved despite PMd lesions and hence such vector-like reach planning must be maintained by other brain regions.

Reach-related areas within the posterior parietal cortex (PPC), namely the parietal reach region (PRR) in the medial wall of the posterior intraparietal sulcus (IPS) of macaque monkeys and its functional human homologue in neighboring parts of superior parietal lobule (SPL), are likely substrates that could subserve vector-like, straight reaching. In fact, monkey PRR and human SPL have been demonstrated to represent reaches in terms of hand-target difference vectors^6,10^, i.e. in an optimal format for coding straight reach paths. Several electrophysiological studies demonstrated that these reach planning regions in PPC may also contain trajectory-related information beyond vector coding. Note, however, that unlike the work on PMd, most of these studies focused on neural activity during reach execution^11–14^ but not on planning. A notable exception is the study of Torres and colleagues^15^, who utilized a simplified obstacle avoidance task. They demonstrated that single cells in monkey PRR modulated their activity prior to the reach whenever a barrier blocked the direct reach path. It was unclear, however, whether the modulation observed in this study truly reflected initial reach direction or, alternatively, strategical chances in initial hand posture present during the planning stage. Taken together, previous research on reach planning in monkey posterior parietal cortex has highlighted its role in the vector-like coding of reach movements. It is unclear, however, whether it also contributes to the planning of complex trajectories.

Here we tried to reveal how trajectory information is represented prior to movement execution in reach-related areas of the human brain, namely areas SPL and PMd. We intended to examine how trajectory representations change when a movement plan could theoretically be constructed by just defining a vector between the initial hand position and a target as compared to situations when these difference vectors are identical but the movement paths vary. Based on previous research we expected to reveal representations of trajectory plans in human SPL^11–16^ and PMd^7,8,16^ (compare above) and, possibly, in primary motor cortex^8,17^ as well as supplementary motor area^8,16^. On the basis of the abovementioned research, we assumed that the trajectory representations in SPL and PMd would likely differ depending on the type of the movement required. Specifically, we hypothesized that while PMd should contribute to the preparation of complex trajectories, SPL would be exclusively engaged in planning straight and direct paths.

## Results

To address our hypotheses, we conducted two human functional magnetic resonance imaging (fMRI) experiments where subjects had to plan and execute finger reaches towards visually cued targets. Two groups of twelve and seven volunteers took part in Experiments 1 and 2, respectively. All of them were right handed, had no history of neurological disease and had normal or corrected to normal vision (see “METHODS” for details). All volunteers gave their written informed consent according to the Declaration of Helsinki prior to the experiment, and the study was approved by the local ethics committee. In Experiment 1 we varied the length of complex (curved) reach trajectories while keeping the hand-target vector constant across conditions. This experiment mimicked situations that enforce the programming of detailed trajectories (like during obstacle avoidance). In Experiment 2 we varied the distance to the target, and thus the length of the hand-target vector, while instructing subjects to perform simple, straight reaches towards it. We expected that if neural representations of reach trajectories are represented prior to movement, increasingly larger neural populations should be recruited to represent certain scalable trajectory properties (i.e. length or complexity), reflecting e.g. the increasing number of intermediate points along the movement path^18^. Therefore the average BOLD signal amplitudes extracted from a given region should increase with these properties as they scale up.

**Figure 1:**
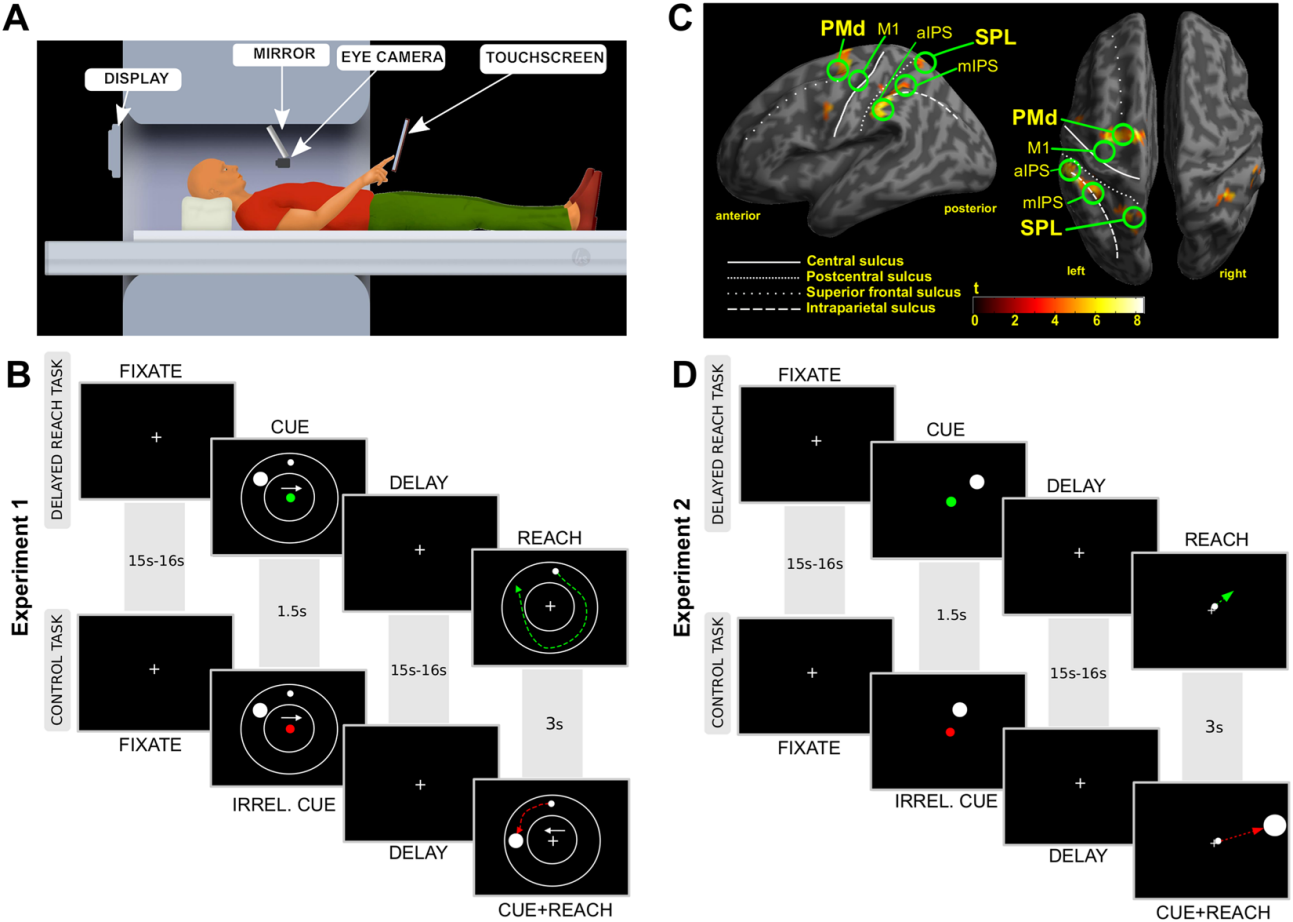
(**A**) MRI-compatible virtual reality reach setup. (**B**) Timeline of the delayed reach task (DRT) and the control task (CT) of Experiment 1. Subjects were supposed to reach the target (filled large circle) by moving their finger from the starting position (filled small circle) to the target in either clockwise or counter-clockwise direction, as was specified by the white arrow cue. These cues were shown in the CUE period of both conditions but were relevant only in the DRT. In the CT all initial cues were irrelevant and the ultimate movement was instructed by a new set of cues presented during the REACH phase. Each condition, DRT and CT, was indicated by its respective color cue, green and red, respectively. Colored dashed lines illustrate putative reach trajectories in both tasks. Additional afterimage-masks presented after both CUE and REACH screens are not shown (see “METHODS” for details). The objects are not plotted to scale. (**C**) Planning Activity. Inflated cortical surface with an overlay of the statistical contrast of delay-related planning activity (DRT > CT) obtained from 12 subjects in Experiment 1 (p < 0.001, uncorrected; t-value > 4.0). Labels identify regions of interest that were included in our ROI analyses. Major anatomical landmarks are labeled in addition. (**D**) Timeline of an exemplary delayed reach (DRT) and control trial (CT) in Experiment 2 (see RESULTS and compare B for details).

For the purpose of our experiments, we constructed a virtual-reality reach environment, consisting of an MR-compatible resistive touch panel and a rear projection display system allowing subjects to receive visual feedback about their reaching finger position in a spatio-temporal correspondence with the true movement (Fig. 1A). Subjects were positioned with their head tilted forward inside the head coil to allow them to naturally look in the direction of their fingertip position although without direct vision of their hand.

Experiment 1 (Fig. 1B) consisted of a circular reaching paradigm that comprised of two task variants. The first variant was a delayed reach task (DRT), which we used to trace reach-trajectory-related activity during planning and execution. The participants were required to remember an initially cued target location (“CUE”-phase), and then, after a delay (“DELAY”-phase), a “go” cue appeared that prompted the participants to move their finger to the now invisible target location (“REACH”-phase). The DRT was contrasted with a second task, namely a control task (CT), in which subjects’ goal was to ignore the initial spatial cue and, after a delay, to move to a visible target presented at a new location. The key difference between both tasks was that in the DRT, the subjects had to plan a movement well before its execution (during the delay epoch)^19^, whereas in the CT, the movement was only planned after the “go” cue appeared, namely when the actual target was presented. The key idea is that during the delay period of the DRT one can assess planning activity in the absence of the sensory cues and before a movement is being executed. By contrasting the respective activity estimates in the CT with the DRT one can further control for processes common to both tasks such as task-unspecific motor preparation^20^.

**Figure 2:**
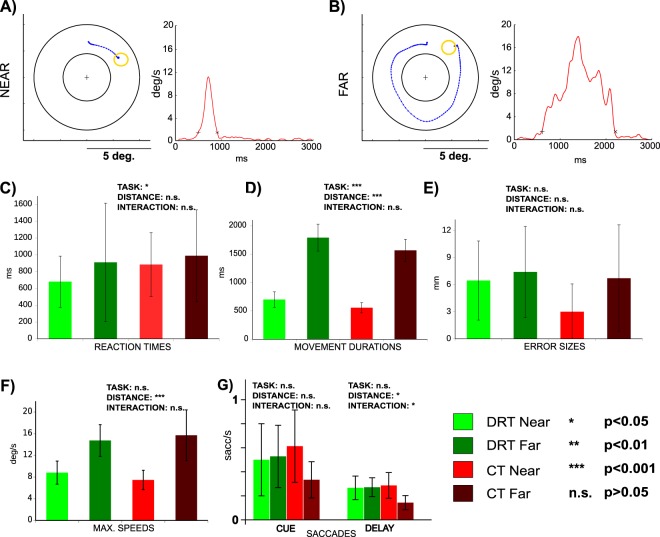
Movement performance in Experiment 1. (**A**,**B**) Exemplary reach trajectories from a single subject (left panels) and the respective movement speed profiles throughout the REACH phase (right panels) for both a “NEAR” (**A**) and a “FAR” (**B**) condition are depicted. (**C**–**G**) The individual panels show our estimates of behavioral performance as a function of “TASK” and “DISTANCE” and report the influence of these factors on these estimates as well as their interaction, as was assessed by two-way repeated measures ANOVAs (n.s. not significant; *p < 0.05; **p < 0.01; ***p < 0.001). (**C**) Reaction times were significantly shorter in DRT than in CT. (**D**) Movement durations were significantly longer in DRT than in CT and longer for “FAR” trajectories than for “NEAR”. (**E**) Error sizes were constant across all conditions. F) Maximal speeds were higher for “FAR” reaches. (**G**) Average frequencies of fixational saccades in CUE an DELAY epochs of respective conditions. Saccades were less frequent in CT “FAR” than in all other conditions. Error bars represent SEM. See RESULTS for detailed statistics.

In both tasks the finger starting location was the topmost position (=0° or 12 o’clock) between two concentric circles, which confined a circular “movement space” between them. The task was indicated by a color cue: a green cue indicating DRT and a red cue indicating CT. The current location of the finger was indicated by a small dot, which was visible during the CUE and the REACH phase only. An arrow cue indicated either a clockwise (right pointing arrow) or a counter-clockwise movement (left pointing arrow) towards the target cue. The target cue was shown at a position within the movement space that was, relative to the starting location (0°), either rotated to the left (by −60° or −40°) or to the right (by 40° or 60°). Accordingly, reaches needed to be executed toward the instructed target in a clockwise or counter-clockwise direction, respectively, and along a circular path within the movement space. Importantly, the combination of target and arrow cue thereby instructed movement paths of varying distance (“NEAR” or “FAR”; see Fig. 1B; also compare Fig. 2A,B). This procedure allowed us to capture trajectory-related information and to isolate it from information related to an initial hand-target vector and an eye-target vector, which both were kept constant in this task (on average across trials). Moreover, this procedure ensured that the target and any retrospective memory thereof would be the same across conditions while reach distance (and complexity) and any related prospective processes engaged in reach planning would vary. In the CT the initial cues were irrelevant and the circular movement was specified by independently selected directional and target cues displayed during the movement epoch (Fig. 1B).

As a first step, we analyzed subjects’ behavior in Experiment 1 in terms of their reaction times as well as the duration, speed, endpoint error of movement and frequency of residual saccades. In brief, 2 × 2 (repeated measures) ANOVAs with the factors TASK and DISTANCE were performed on subjects’ average behavioral estimates. The respective statistical analysis (Fig. 2C) yielded significantly shorter reaction times in the DRT condition than in the control condition, indicating that the movements were actually pre-planned in the DRT (factor TASK: df = 11, F = 6.8, p = 0.024, eta^2^_G_ = 0.0207; all other effects were not significant: DISTANCE: df = 11, F = 2.5, p = 0.140, eta^2^_G_ = 0.0287; TASK*DISTANCE: df = 11, F = 1.5, p = 0.252, eta^2^_G_ = 0.0041)^19^. Movement durations were significantly longer in DRT (TASK: df = 11, F = 27.7 p = 0.0003, eta^2^_G_ = 0.235) and for longer trajectories (DISTANCE: df = 11, F = 701.4, p < 0.0001, eta^2^_G_ = 0.908). It is noteworthy that the latter effect was driven by much larger duration differences (see Fig. 2D). There was no interaction between the two main factors (TASK*DISTANCE: df = 11, F = 1.7, p = 0.21, eta^2^_G_ = 0.014). Endpoint error (see Fig. 2E) was constant across tasks and distances (TASK: df = 11, F = 2.4, p = 0.148, eta^2^_G_ = 0.050; DISTANCE: df = 11, F = 3.5, p = 0.087, eta^2^_G_ = 0.062; TASK*DISTANCE: df = 11, F = 3.1, p = 0.104, eta^2^_G_ = 0.023). Maximal movement speed (Fig. 2F) did not differ across tasks (TASK: df = 11, F = 0.14, p = 0.72, eta^2^_G_ = 0.001). It was however higher for longer trajectories (DISTANCE: df = 11, F = 57.87, p = 0.00005, eta^2^_G_ = 0.589). The interaction effect was not significant (TASK*DISTANCE: df = 11, F = 2.71, p = 0.13, eta^2^_G_ = 0.037). Finally, the frequency of saccades (Fig. 2G) was indistinguishable between DRT and CT in the CUE phase (TASK: df = 9, F = 0.14, p = 0.72, eta^2^_G_ = 0.00061; DISTANCE: df = 9, F = 0.23, p = 0.64, eta^2^_G_ = 0.00169; TASK*DISTANCE: df = 9, F = 1.85, p = 0.21, eta^2^_G_ = 0.01130), and was lower for CT “FAR” reaches than in all other conditions in the DELAY phase (TASK: df = 9, F = 2.8, p = 0.127, eta^2^_G_ = 0.010; DISTANCE: df = 9, F = 6.1, p = 0.035, eta^2^_G_ = 0.039; TASK*DISTANCE: df = 9, F = 7.2, p = 0.025, eta^2^_G_ = 0.021). Most importantly, the saccade rates in both CUE and DELAY phases of DRT did not differ for our trajectory conditions (“NEAR” vs. “FAR”).

Subjects’ task-related brain activity was assessed with fMRI. Experiments were performed in a 3 T Siemens Trio scanner. Functional imaging was done using EPI sequences with 2 s temporal resolution and 3 × 3 × 4 mm voxel size. Functional data were analyzed using SPM8 and were modeled using a general linear model, in which we included the following regressors of interest: the main epochs of a trial (“CUE”, “DELAY”, “REACH”) were modeled separately for each experimental task (DRT vs. CT) and for each trajectory length (“NEAR” vs. “FAR”). In order to assess correlates of trajectory planning in SPL and PMd we chose a region of interest- (ROI-) based approach. In the first step we delineated a set of brain regions recruited in movement planning by contrasting delay epochs of DRT and CT. This was done by contrasting activity estimates during the delay epochs of DRT vs. CT both within the group and in each individual. Single subjects statistical contrasts combined with anatomical criteria were used to adjust the individual ROI selection in order to account for inter-individual differences in functional brain organization (see “METHODS” for details).

Figure 1C and Supplementary Fig. S1 depict the resulting statistical parametric map of the group analysis, exhibiting planning regions. In Fig. 1C we highlighted our *main ROIs*, namely PMd and SPL, as previous research demonstrated their involvement in reach trajectory coding (see introduction). For the sake of completeness we included other areas engaged in hand movement planning: anterior and middle intraparietal sulcus (IPS) and supplementary motor area (SMA). We considered these latter areas as *complementary planning ROIs*. In addition we included primary motor cortex (M1) due to its potential engagement in trajectory representation^8^, as well as primary visual cortex (V1), which served as a *control ROI* allowing us to monitor task-unspecific brain activity reflecting visual stimulation during all trial phases. From every ROI we next extracted timecourses of BOLD-signal changes throughout a trial at 1 s temporal resolution. Within each individual we then separately averaged timecourses for each experimental condition. Statistical comparisons were performed across subjects’ average timecourses and between experimental conditions. Activity-timecourses were compared for trajectories of varying length/complexity and separately for each condition. Specifically, we engaged a time-resolved analysis by recruiting multiple paired t-tests performed separately for each time point. We decided on the ROI-based time-course analysis to be able to scrutinize the dynamics of activity changes in planning areas as we expected those to potentially reflect trajectory plan representations. Similar to previous research^21^, the activity maps in our subjects were clearly contra-lateralized with respect to the reaching hand (see Fig. 1C). Accordingly, we focused in our analyses only on these left-hemispheric areas.

**Figure 3:**
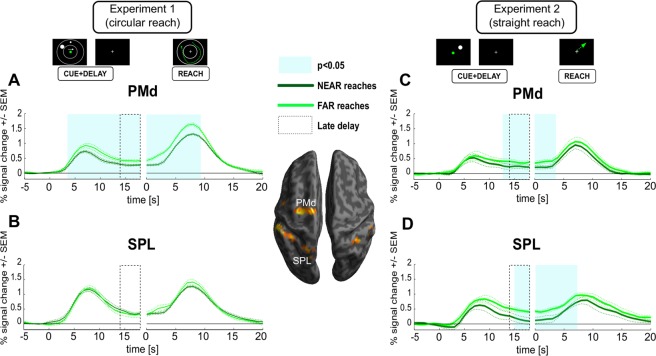
ROI timecourses extracted from PMd (**A**,C) and SPL (**B**,**D**), comparing the fMRI-signal in the delayed reach task of Experiments 1 and 2 (**A**,**B** vs. **C**,**D**, respectively). Cyan-shaded areas represent time epochs during which paired t-test comparisons of signal amplitudes between “NEAR” and “FAR” reaches revealed statistically significant differences (p < 0.05) for at least three neighboring time-points. Such differences were considered indicative of an influence of trajectory. PMd shows different planning-related signal amplitudes in both experiments (leftward part of the panels A and C, aligned to CUE onset). SPL shows trajectory planning signal modulation in Experiment 2 only (**D**). PMd shows significant modulation in the reach phase in both experiments (A and C, right panels, aligned to REACH onset), whereas SPL shows such statistically significant difference only in Experiment 2 (**D**) although a hint of the same effect might be present in the reach phase of Experiment 1 as well (**B**). Dotted gray boxes indicate the late delay phase, in which activity merely represents planning but no longer CUE-related activity. Activity estimates of this period were also used for a subsequent statistical comparison between PMd and SPL across experiments (see RESULTS).

**Figure 4:**
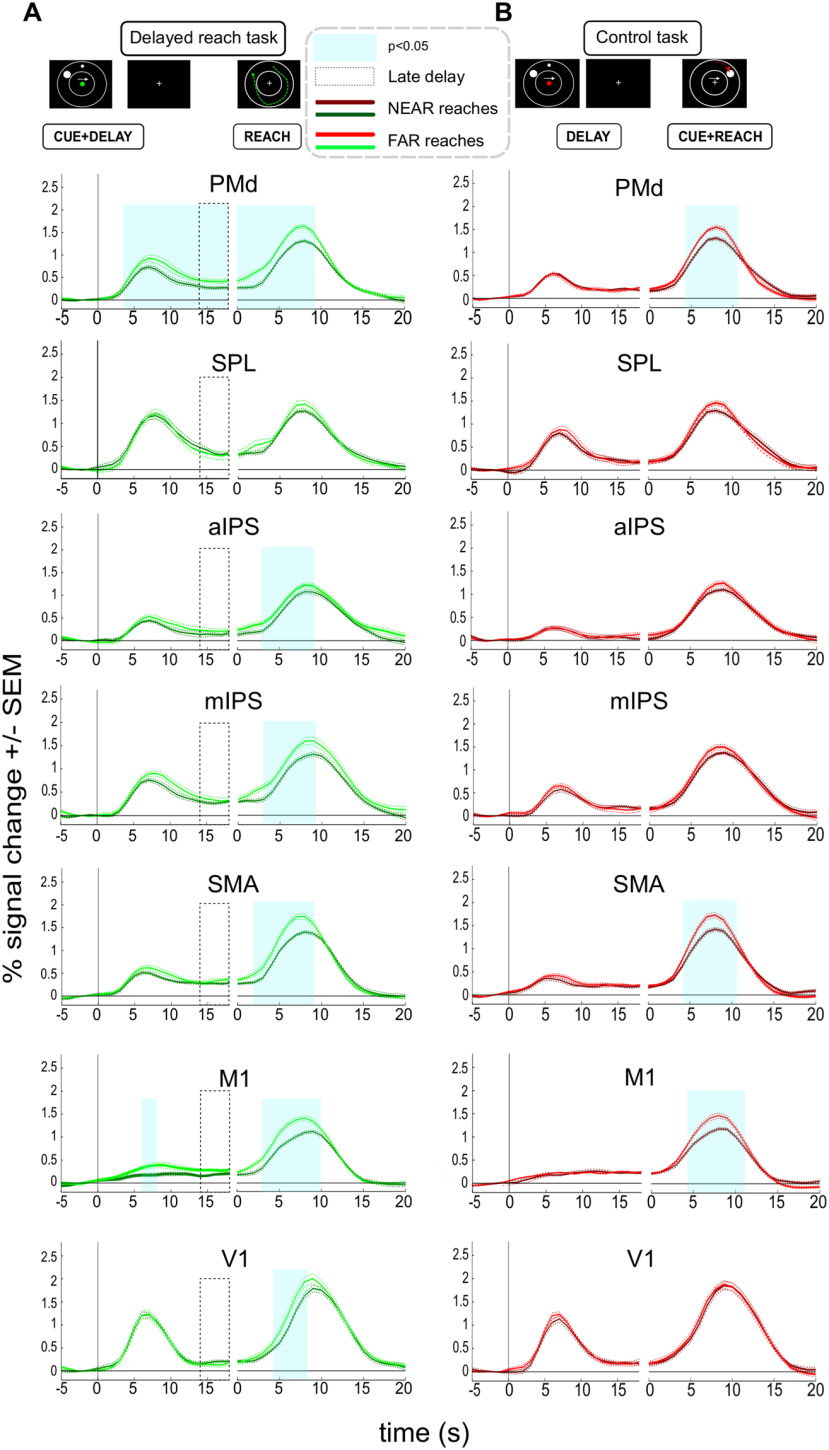
Timecourses of fMRI signals extracted from ROIs in the delayed reach (**A**) and control tasks (**B**) in Experiment 1. Left panels are aligned to CUE onset while right panels are aligned to REACH onset. Cyan-shaded areas represent time epochs during which paired t-test comparisons of signal amplitudes between “NEAR” and “FAR” reaches revealed statistically significant differences at p < 0.05 for at least three neighboring time-points. Such differences were considered indicative of an influence of trajectory. In the DRT both PMd and M1 showed transient trajectory representation during the planning stage (leftward part of the panels, aligned to CUE phase onset). PMd, M1, SMA, mIPS, aIPS and V1 showed differences between the two types of trajectories during the reach stage (rightward part of the panels, aligned to REACH phase onset). (**B**) No ROI shows planning-related differences in the CT. As in the DRT, PMd, SMA and M1 exhibit execution-related differences.

Figure 3A,B show respective timecourses (averaged across subjects’ means) that were obtained during the DRT task for both main ROIs (A: PMd; B: SPL). The leftward part of each panel depicts the timecourses aligned to CUE onset while the rightward part represents the same timecourses but aligned to the onset of the REACH-phase. Note that we assume a typical (5–6 seconds) delay in time to peak of the event-related haemodynamic response in the human brain^22,23^. Changes in planning activity in the absence of any residual CUE-related activity can, accordingly, be directly inspected during the late DELAY-phase (the last 4 s of the delay period, indicated by dashed boxes in Figs 3, 4 and 6), when all the activity related to visual target and cue processing is absent (compare V1 activity in Fig. 4) and when activity is not yet affected by movement execution. During this time period we observed a significant increase in BOLD activity during planning of longer/more complex trajectories in PMd (Fig. 3A; cyan-shaded area). Note that this difference emerged already early after cue presentation and already then might have reflected a trajectory-related difference in planning. However, as was pointed out before, additional CUE-related modulations of the fMRI-signal can – even if unlikely – not be completely ruled out. Moreover, the difference between conditions was also present during the REACH-phase. Note, however, during this period, signal modulations are contaminated by systematic differences between conditions such as movement duration or speed (compare the results of our behavioral analyses) and the related differences in visual movement feedback. In contrast to PMd, trajectory-related signal modulation was virtually absent in SPL in both trial phases (Fig. 3B). Finally, we did not observe any trajectory-related variation of BOLD-signals in the DELAY phase of the control task in either of our main ROIs (Fig. 4B). Also in the REACH-phase PMd exhibited a significantly higher signal amplitude during FAR as opposed to NEAR reaches (Fig. 4B). As was mentioned before for the DRT, this activity pattern is likely accounted for by the systematic differences in movement execution and movement feedback.

In none of our additional ROIs we could reveal a significant signal-difference between NEAR and FAR during the late DELAY-phase. It is noteworthy, however, that in M1 we also observed a significant effect of trajectory but only early during the DELAY-phase (Fig. 4A). Similar to PMd we observed an effect of reach trajectory during reach execution in V1, M1, SMA, mIPS and aIPS (rightward panels of Fig. 4A). In all cases activity was higher for the more complex/longer trajectory. Note, again, that the presence of these effects is not necessarily related to planning. It might be rather explained by the systematic differences in movement and - as is clearly indicated by V1 activity - by the respective amount of visual motion that we provided as feedback about subjects’ movements. As was true for PMd and SPL, we did not see any trajectory-related variation of BOLD-signals in the DELAY-phase of the control task in either of the additional ROIs, but only during the REACH-phase (Fig. 4B).

In summary, the results of Experiment 1 are consistent with the idea that PMd – and potentially also M1 – represent plans for upcoming reach trajectories. Planning activity reflected differences in the length of curved trajectories despite the initial hand-target difference vectors were identical across trials. In the next experiment, i.e. Experiment 2, we scrutinized planning activity from the same ROIs in a situation in which movements were directed straight towards target and, thus, could – at least potentially – be defined by a hand-target difference vector. In other words, there was no explicit need to represent trajectories during movement planning.

**Figure 5:**
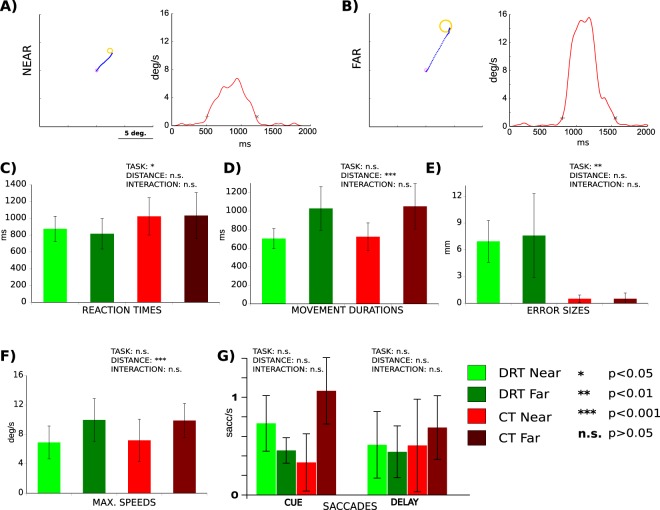
Movement performance in Experiment 2. (**A**,**B**) Exemplary reach trajectories of a single subject (left panels) and the respective speed profiles (right panels) for both a “NEAR” (**A**) and a “FAR” (**B**) condition. (**C**–**G**) The individual panels show our estimates of behavioral performance as a function of “TASK” and “DISTANCE” and report the influence of these factors on the respective estimates as well as their interaction, as was assessed by two-way repeated measures ANOVAs (n.s. not significant; *p < 0.05; **p < 0.01; ***p < 0.001). Error bars represent SEM. (**C**) Reaction times were significantly shorter in DRT than in CT. (**D**) Movement durations were significantly longer for “FAR” trajectories than “NEAR”. E) Error sizes were larger for DRT. (**F**) Maximal speeds were higher for “FAR” reaches. See RESULTS for detailed statistics. (**G**) Average frequencies of fixational saccades in CUE an DELAY epochs of CT and DRT. The rates of fixational saccades were indistinguishable across all conditions.

The overall design of Experiment 2 was similar to the one used in Experiment 1 in that we contrasted a delayed reach planning task with a direct reach task. This time, however, we used a simple center-out reaching task (compare Fig. 1D) in order to see whether the potential trajectory-related scaling of the BOLD-signals would be visible in brain activity even if a given reach trajectory could be defined by a simple difference vector between target and hand, as such vector-based programming has been suggested by behavioral findings^24–26^ (but compare^27^). We manipulated reach amplitude by positioning the targets at two different distances and at randomly chosen radial positions in the upper-right quadrant of the visual field (see Fig. 5A,B for examples). The idea behind this manipulation was to additionally uncover potential trajectory representations for simple, straight reach plans, and, as in Experiment 1, we expected that planning of longer trajectories should result in higher BOLD signal amplitudes (see INTRODUCTION).

Similar to Experiment 1, reaction times (Fig. 5C) were significantly shorter in the DRT (“TASK”: df = 6, F = 7.8, p = 0.031, eta^2^_G_ = 0.0039) suggesting that subjects preplanned their movements in this condition. All other effects were not significant (“DISTANCE”: df = 6, F = 1.2, p = 0.322, eta^2^_G_ = 0.1786; “DISTANCE*TASK”: df = 6, F = 1.9, p = 0.220, eta^2^_G_ = 0.0073). As in Experiment 1, movement durations (Fig. 5D) were significantly longer for longer trajectories (“DISTANCE”: df = 6, F = 42.8374, p = 0.00061, eta^2^_G_ = 0.45535). All other effects were not significant (“TASK”: df = 6, F = 0.5772, p = 0.47619, eta^2^_G_ = 0.00360; “DISTANCE*TASK”: df = 6, F = 0.0014, p = 0.97177, eta^2^_G_ = 0.00001). The endpoint error sizes (Fig. 5E) were significantly higher in the DRT (“TASK”:df = 6, F = 34.17, p = 0.0011, eta^2^_G_ = 0.6545). This difference likely resulted from lower precision of memory- vs. visually-guided reaches. Most important for our study, both the main factor “DISTANCE” and its interaction with”TASK” were not significant (“DISTANCE”: df = 6, F = 0.20, p = 0.6735, eta^2^_G_ = 0.0045; “DISTANCE*TASK”: df = 6, F = 0.13, p = 0.7326, eta^2^_G_ = 0.0045). Maximal speeds (Fig. 5F) were significantly higher for longer trajectories (“DISTANCE”: df = 6, F = 77.86, p = 0.00012, eta^2^_G_ = 0.26356). All other effects were not significant (“TASK”: df = 6, F = 0.20, p = 0.67151, eta^2^_G_ = 0.00043; “DISTANCE*TASK”: df = 6, F = 0.47, p = 0.51874, eta^2^_G_ = 0.0014). Finally, saccade frequencies (Fig. 5G) were not different across conditions both in the CUE (“TASK”: df = 4, F = 1.1, p = 0.358, eta^2^_G_ = 0.043; “DISTANCE”: df = 4, F = 4.8, p = 0.093, eta^2^_G_ = 0.090; “DISTANCE*TASK”: df = 4, F = 4.1, p = 0.114, eta^2^_G_ = 0.112) and in the DELAY phase (“TASK”: df = 4, F = 0.91, p = 0.39, eta^2^_G_ = 0.0041; “DISTANCE”: df = 4, F = 0.51, p = 0.51, eta^2^_G_ = 0.0034; “DISTANCE*TASK”: df = 4, F = 1.50, p = 0.29, eta^2^_G_ = 0.0048).

We next considered task-related changes of brain activity in our main and in the complimentary planning-related ROIs. Note that we used a similar procedure for ROI selection to the one used in Experiment 1. The actual brain regions selected for further ROI analyses were practically the same as in Experiment 1 (see Supplementary Fig. S1, compare also METHODS for further details on ROI selection).

**Figure 6:**
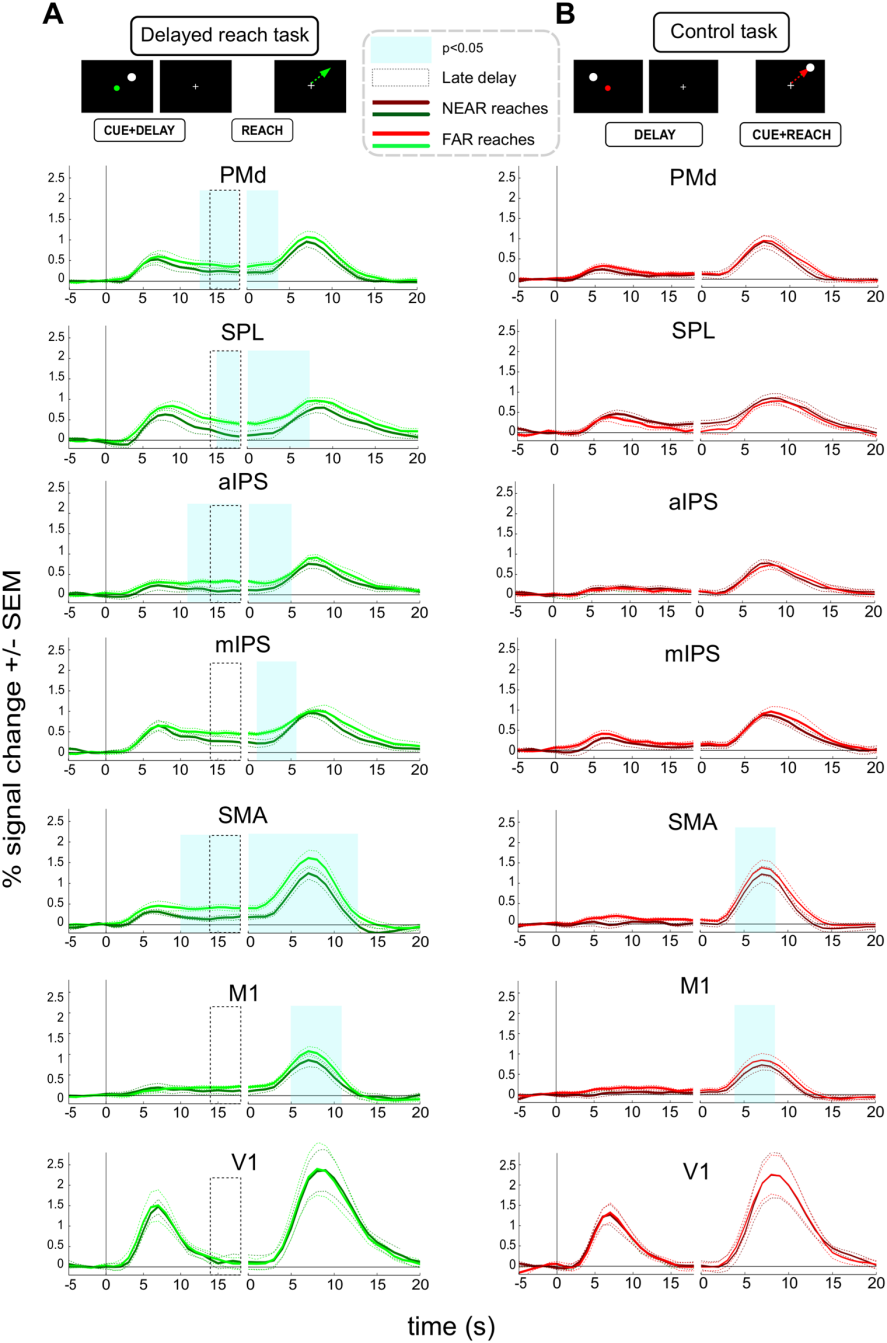
Timecourses of fMRI signals extracted from ROIs in the delayed reach and control tasks in Experiment 2. Left panels are aligned to CUE onset while right panels are aligned to REACH onset. Cyan-shaded areas represent time epochs during which paired t-test comparisons of signal amplitudes between “NEAR” and “FAR” reaches revealed statistically significant differences at p < 0.05 for at least three neighboring time-points. (**A**) PMd, SPL, SMA and aIPS show significant signal differences during the planning epoch in DRT. All areas except V1 show differences during the reach epoch. Note that some of these areas show differences only before the reach execution-related peak of the BOLD response, suggesting that some of these differences might still refer to planning during the late delay. (**B**) In the control task, no ROI showed planning-related differences. However, both SMA and M1 exhibited differences during the reach stage (also compare to A and to Fig. 4).

The BOLD signals in these ROIs during the reach phase of the DRT were quite similar to those observed in Experiment 1: longer trajectories yielded larger signal amplitudes in PMd, SPL aIPS, mIPS, SMA and M1 (see Figs 3C,D and 6A, rightward part of panels). More importantly, for our main ROIs the planning-related BOLD signals extracted during the late DELAY phase of the DRT were markedly higher for longer trajectories not only in PMd but this time in the SPL too (Fig. 3C,D; compare time period indicated by the dashed box in the leftward part of each panel). Higher delay-related BOLD signals for longer trajectories were also observed in two of our complimentary ROIs: SMA and aIPS (Fig. 6A). No planning-related signal modulation was observed in M1 or in any other additional ROI. In the control task, no ROI showed any trajectory-related activity during the DELAY phase (Fig. 6B). Only during the REACH phase, M1 and SMA exhibited a modulation of the BOLD-signal as a function of trajectory (Fig. 6B). This resembled their respective signal changes during the REACH phase in DRT (Fig. 6A) and likely can be attributed to the systematic differences in movement execution (also compare Experiment 1). The lack of similar V1 modulation likely resulted from the much lower amount of visual motion as evident from the smaller movement duration differences between trajectories (Fig. 5D).

In order to directly test for the differences in the activation patterns in our main ROIs, SPL and PMd, across the two experiments, we performed an additional mixed model ANOVA with the factors “EXPERIMENT”, “DISTANCE” and “ROI”, comparing the activity estimates of the late delay phase of the DRT trials. These estimates captured the average activity during the last four seconds of the DELAY phase (see dashed boxes in Fig. 3). The analysis revealed a significant three-way interaction (F = 7, df = 17, p = 0.017, eta^2^_G_ = 0.03), further confirming that SPL and PMd exhibited distinct patterns of planning activity in both tasks, namely a stronger contribution of PMd to the planning of complex trajectories in the DRT of Experiment 1 as compared to SPL, while both areas exhibited signal differences representing the straight, vector-like movement trajectories in the DRT of Experiment 2.

Finally, to further scrutinize the apparent differences in activations between the PMd and SPL across experiments, we calculated Bayes factors for each of these ROIs in both experiments (see METHODS for details; see also Supplement 1 for comparisons of the complimentary ROIs). We added this analysis, as our conventional statistic did not control for type II errors as strictly as for type I errors(compare^28^). Specifically, performing Bayes factor analyses should allow us to judge whether any DRT-related planning activity estimates of our primary ROIs during the late delay (compare dashed boxes in Fig. 3) are the same for trials with different trajectory length (null hypothesis) or whether they are different (alternative hypothesis). This is particularly relevant in case of Experiment 1, namely to reveal whether in case of SPL there is substantial evidence in favor of the null-hypothesis, i.e. that activity is indistinguishable across NEAR and FAR conditions that have identical hand-target vectors but that vary in terms of the length/complexity of trajectories. A Bayes factor value below 0.33 would thereby provide strong evidence for a lack of signal differences while values above 10 would indicate strong evidence in favor of signal differences between NEAR and FAR conditions^28^. Bayes factors for Experiment 1 equaled 60.79 for PMd and 0.22 for SPL. This result is consistent with a role of PMd in planning complex reach trajectories of varying length. More importantly, there is strong evidence that planning activity in SPL did not differ for curved trajectories that varied in length but shared identical hand-target vectors. In Experiment 2 the Bayes factors were 10.34 for PMd and 201.29 for SPL. The strong evidence in favor of signal differences parallels previous statistical analyses and shows trajectory representations in both main ROIs during straight reach planning.

## Discussion

In Experiment 1 we showed that different reach trajectories for targets kept at the same visual locations produce differential planning responses in dorsal premotor cortex but not in SPL, where responses were indistinguishable. Experiment 2 allowed us to further demonstrate that trajectories are represented in PMd even if reaches could, at least in principle, be coded by a simple hand-target difference vector. Moreover, we show that, like in case of PMd, the activity was modulated by the trajectory of straight reaches in the medial portion of SPL too. Comparing the results from these two experiments, we may note that while PMd contains representations of trajectories irrespective of their complexity, SPL primarily encodes trajectory plans for simple reaches directed straight towards a target.

We ensured that the reported differences could not be accounted for by subjects’ residual eye movements (Figs 2G and 5G). Moreover, constant error rates across conditions, as were present in both experiments (Figs 2E and 5E), suggest that the different planning-related signals did not simply result from increasing task difficulty, but rather reflected parameters of planned trajectories. The particular design of Experiment 2 further ensured that such differences in task difficulty between “NEAR” and “FAR” should not arise in the first place (compare METHODS). Finally, in Experiment 1 we instructed the same target locations (across trials and conditions) while varying the way to the target (i.e. the trajectory). This allowed us not only to keep initial eccentricity/direction of target location balanced across conditions but this also guaranteed that any attention towards the target locations (or cues), or any retrospective memory thereof, would likewise be identical across tasks. Hence, the reported differences in brain activation should exclusively relate to the process of planning different reach trajectories.

Does reaching always require planning of trajectory? One alternative possibility, suggested by prior literature, is that a reach is initially defined by a vector pointing either towards the final target location or, alternatively, towards the initial direction of movement^7^. Then, during reach execution, the hand would be guided on-line by a feedback-based control system^24–26^, allowing even for guiding more complex shaped reaches^26^. This way, only the first desired state (goal) and not the whole trajectory would need to be planned in advance. As an alternative to the above, it may be hypothesized that the reach trajectory is defined and represented at the initial stages of reach planning^18^ and only then, this initial plan is being converted to respective motor commands during movement execution while likewise allowing for on-line corrections for potential movement inaccuracies. Most previous research on trajectory coding concentrated on the movement execution stage, albeit with some exceptions which (also) focused on reach planning^7,8,15^. On the basis of the electrophysiological results provided by these studies, however, one could not determine whether the changes in planning activity reflect any global trajectory parameter such as the overall movement path. Conversely, they could also represent only initial components of trajectory like the initial hand posture^15^ or the initial movement direction enforced by additional cues (e.g. obstacles)^7,8^. These latter studies, in which representations of such specific parameters were reported, the remaining parts of movement planning and execution could still be guided by the aforementioned on-line control system. For instance, findings of Pearce and Moran show that the population activity in PMd seemingly encodes the initial direction of an upcoming movement regardless of the target position. Yet, based on their results one cannot infer anything about representation of the remaining parts of trajectory^7^. Hence, the currently available physiological data leave it open whether or not there is an explicit neural coding of global trajectory parameters during reach planning. Our results, in turn, seem to support the idea that PMd does code global, scalable trajectory parameters prior to a movement. We exhibited a modulation of the averaged BOLD response during planning of varying reach trajectories despite target directions, initial movement directions, and hand-target vector lengths were, on average, constant. PMd activity represented trajectory information not only when the situation required the precise programming of a more complex movement path (Experiment 1), but even if planned trajectories were straight and direct (Experiment 2). This highlights a vital and general role of premotor cortex in trajectory planning.

Given the nature of our experimental design and of our recording methods, however, we cannot further detail the precise global trajectory parameters underlying the signal changes that we revealed. Yet, as we have already argued above, it is at least highly unlikely that the observed signal differences can be explained by specific spatial parameters such as initial difference vector or obstacle location (also compare our discussion in the next paragraph). Due to the relatively coarse spatial resolution of our BOLD signal recordings (see “METHODS”), the activity estimates averaged within the regions-of-interest covered large neural populations. This approach allowed us to avoid the problem of measuring only specific correlates of trajectory (such as initial direction), but, in turn, did not allow us to attribute the changes in planning activity to any of the specific kinematic parameters correlated to the trajectory length, such as reach durations, numbers of intermediate segments/points or exact path shapes. Hence, while we were able to assess differences in the global “representation” of trajectory plans by the underlying neural population, our approach did not allow us to attribute the changes in planning activity to any of the specific kinematic parameters that are reflected in the firing patterns of individual neurons. These detailed properties of trajectory planning have to be addressed using more sensitive methods. Our findings also leave open the question about the actual local topographic distribution of trajectory coding neural populations within the ROIs. The earlier electrophysiological findings of Messier and Kalaska, who reported that individual PMd neurons code both reach amplitude and direction, may present important hints to address this issue^29^.

Similar to PMd, superior parietal lobule activity increased during the planning of straight, direct reaches towards more eccentric targets in Experiment 2. Contrary to PMd, however, SPL planning activity did not differentiate between the more complex trajectories of Experiment 1, where the hand- and eye-to-target initial difference vectors were kept equal regardless of trajectory. These results are consistent with the notion that SPL could chiefly represent straight, vector-like “default” reach plans, which would be indistinguishable under conditions like in Experiment 1, where these default hand-target vectors had the same length in both planning conditions and, therefore, would not lead to any difference in BOLD-signal amplitudes. One might further ask whether SPL thereby indeed encoded an initial difference vector (similar to saccades) or the straight trajectory defined along such vector, as the two features were inherently correlated in Experiment 2. As suggested by anatomical studies, SPL (and other parietal regions) has a relative over-representation of the visual periphery as compared to visual areas^30–32^ (but see^33^ for alternative view). The mere coding of the difference vector could, accordingly, recruit larger neural populations representing more peripheral targets, and potentially lead to an increase in the total BOLD signal for more eccentric target locations in Experiment 2. However, as was demonstrated by Kimmig *et al*. in their study on saccades^34^, the coding of larger difference vectors (or rather topological coding of more eccentric target locations) themselves is not sufficient to modulate the amplitude of the BOLD signal in the way we observed here, as it probably does not require recruiting increasingly larger neural populations the longer the saccade vector. The same argument likewise accounts for movement durations (as the larger saccade amplitude, the longer its duration). Clearly, while the cortical planning of reaches and saccades is largely distinct, it still does share several common principles. It is therefore hard to see why the chosen target distances and movement durations alone should lead to gross amplitude differences in the BOLD response, especially given the fact that comparable parameters did not lead to BOLD signal differences in the aforementioned saccade planning task. Adding to this, even though some data suggest existence of a relative over-representation of the periphery, there is still an absolute over-representation of the central visual field in posterior parietal cortex, predicting a weaker representation of more peripheral locations^33^. For these various reasons we reject the possibility that in Experiment 2 the movement plans were coded on the basis of difference vector alone. In our view, the observed increase in SPL activity for more eccentric reaches in Experiment 2 is consistent with the idea that the region’s role is indeed to represent a target location but, moreover, to initially construct a “simple” trajectory aimed directly at the target (e.g. through interpolated intermediate points^18^). It well might be that such direct and straight trajectory representation is encoded by neural populations in SPL by default, no matter what sort of movement path is actually required in a given context. This, in turn, could explain why monkeys with lesions of premotor cortex and intact posterior parietal cortex cannot plan complex hand trajectories that would allow them to effectively avoid obstacles. Instead, these monkeys still try to reach straight towards targets and bump into obstacles^9^. Moreover, recent findings showed that PPC inactivation in monkeys impairs their ability to reach along straight paths and the reaches become significantly curved^35^. Such automatic planning of simplified, straight trajectories by SPL could be useful in various everyday situations and crucial whenever a rapid response is required (e.g. when swatting a fly). We admit, however, that there may still exist differences between the straight movements executed in this experiment and certain other fast, reflexive hand movements performed in more natural settings, where adhering to the flat surface of a touch-panel is not required.

Given the obvious difference in movement plan representation between PMd and SPL, as was described here, one could conceive a hierarchical model in which an initial trajectory plan is formed in SPL based on the difference vector pointing directly towards the target location. As information transfer from posterior parietal cortex to M1 is faster than to PMd^36^, the simple movement plan may be quickly put into action. If required, this initial plan is “overwritten” by other frontal areas (such as PMd), which possibly consider additional spatial constraints that would interfere with execution of the reach along the initially defined, direct path - like information about obstacle locations^7,15,20^. PMd might incorporate such additional information to construct an ultimate (potentially more complex) trajectory plan, which is passed on to areas responsible for its further processing and execution (like M1). In fact, as we already described above, lesions to premotor cortex of macaque monkeys make them unable to avoid obstacles or, alternatively, to update their initial motor plan^9^. In addition, PMd has been shown to represent those motor plans that are actually selected for execution, rather than all the possible ones. This does further imply a role of PMd in forming the ultimate trajectory^37,38^. It is worth noting that several authors postulated that PMd may additionally play a governing role in the sensorimotor system, modifying motor plans as required by given context^39,40^. Such detailed hierarchy amongst the cortical areas engaged in planning reach trajectories could not be assessed on the basis of our experiments. The nature of the BOLD signal would not allow distinguishing incoming neural signals from local ones^41^ - a distinction which is critically needed to establish hierarchy. Moreover, such distinction is perhaps particularly challenging when considering posterior parietal and premotor areas that are linked by single-synapse pathways^42–46^. To further detail how exactly trajectory information is represented and transferred throughout the network of areas engaged in sensorimotor processing, causal methods could be utilized in the future to disrupt information flow between specific regions.

In accordance with earlier studies, we did observe trajectory information encoded in M1^47^ activity during movement execution^8,16,17^. The results revealed in Experiment 1 further suggest that M1 might encode trajectory information already at the very early stages of planning reaches along complex paths. In Experiment 2 we did not observe any M1 modulation of this kind, possibly because in this experiment trajectories did not differ with respect to their complexity. The overall finding suggests that only trajectories of greater complexity may require engagement of M1 well before movement execution.

Another interesting finding is the involvement of supplementary motor area in the planning of straight but not of circular movements (see Supplement 1). This result parallels earlier findings of Hocherman and Wise^8^ who also reported more SMA neurons involved in coding straight than curved reach paths, as evident from the number of neurons responding to either of those. Hence, similar to SPL, SMA seems to be more involved in coding direct, straight trajectories. Yet, it still remains to be determined what exact role the SMA plays in this process and whether our observation can be confirmed.

Our study suggests that reach trajectory information is globally represented in premotor and posterior parietal areas of the human brain well before movement execution. Moreover, we reveal differences in the representations of planned reach trajectories across these areas. Specifically, dorsal premotor cortex can seemingly encode both simple and complex reach trajectories while posterior parietal cortex (and possibly supplementary motor area) rather represent plans for movement along simplified, straight and direct paths. Such a parallel and distinct representation of two fundamentally different types of trajectory plans would clearly ask for a meaningful functional interpretation. It is conceivable that emergence of two complimentary reach planning subsystems is desirable from an ecological point of view by offering a high degree of flexibility in adjusting hand movement control to situational demands. This way a parietal subsystem could allow to rapidly reach straight towards an object, whereas a frontal subsystem would take over whenever movements have to be performed with more finesse and when moving along the right path is an integral part of the motor goal.

## Methods

### Participants

Twelve healthy, right-handed participants (11 females) in the age range of 20–32 years (mean age 25 years), participated in Experiment 1. Seven healthy, right-handed volunteers (6 females, age range 20–31 years, mean age 25 years) took part in Experiment 2. Out of these, five subjects had also participated in Experiment 1 (two of them had completed Experiment 2 first). The over-representation of female subjects in both experiments resulted from spatial constraints given our setup (especially touchscreen size and its position, see Fig. 1A), which required particularly slim subjects. Building on results from a similar planning task, which also engaged a delayed response paradigm, we expect that this gender imbalance should not have any qualitative impact on our findings^48^. All participants gave written informed consent prior to participation in the study. All experimental procedures were carried out in accordance with the declaration of Helsinki, and the study was approved by the ethics committee of the University Hospital and the Faculty of Medicine at the University of Tuebingen.

The number of participants was guided by a power analysis (power = 0.80; alpha = 0.05) that was informed by the descriptive statistics of a timecourse analysis on a previously published, similar fMRI dataset. In that study, planning activity varied as a function of movement sequence length^20^. For the power analysis we considered the within-subject activity difference during the late delay period (last 4 sec) in left PMd, namely for a delayed response task that required the planning of a less complex (2 targets) vs. a more complex (4 targets) movement sequence. This analysis suggested a sample size of 11 subjects (two-tailed tests). For Experiment 2 we relaxed this criterion (one-tailed tests), as we had a directional hypothesis (the stronger the activity the more complex trajectory planning). Note that here we measured each experimental condition 20 times per individual, while the study that informed our power analyses only comprised of 9 repetitions per condition.

### MR-compatible reach setup

We realized our experiments in a custom made MRI-compatible virtual reality reach setup, in which we could record 2D movements of subjects’ right index finger and could provide subjects a virtual visual representation of their finger on a stimulus screen (see Fig. 1A). Specifically, visual stimuli were projected via an LCD projector onto a translucent screen, mounted directly behind the head coil of the scanner (1024 × 768 pixels; 60 Hz refresh rate). Subjects viewed the stimulus screen via a mirror, positioned in front of the participant. Viewing distance was approximately 82 cm and roughly matched the distance from participants’ eyes to the touchscreen. To track subjects’ finger movements we used a MRI-compatible motion capture system, utilizing a resistive touchscreen panel from MAG (www.magconcept.com), mounted on a plastic board. This touchscreen-board was placed on top of a plastic rack onto which the stimulus-mirror, a camera for eye movement recordings and the display screen were mounted in addition. Limited by the spatial constraints of the scanner environment, we always tried to approximate a parallel alignment between the touchscreen and the display to guarantee approximate spatio-temporal correspondence between a subject’s finger position and their perceived visual feedback thereof. Subjects were positioned with their head tilted forward inside the scanner head coil, so that they could directly look towards their pointing finger. Ultimately, direct vision of the hand was blocked by both the mirror and additional masks and subjects had to rely on the virtual visual feedback about their finger position instead. All reaches were performed in darkness and the only visual information provided was the one projected through the display system. In order to minimize potential disturbances of the magnetic field by hand and arm movements we stabilized each subject’s arm, elbow and shoulder with foam cushions and adhesive tape, so that only wrist and finger movements were made possible. To minimize movement friction on the touchscreen we had subjects wear a cotton glove on their reaching hand.

Each of our experiments was preceded by a training session during which the subjects familiarized themselves with the tasks demands. All subjects were additionally required to practice the experiment for a minimum of 10 minutes inside the scanner once the MRI setup had been completed.

### Eye recordings

Eye fixation was monitored at 50 Hz sampling rate with an MR-compatible combined camera and infra-red illumination system (MRC Systems) using the ViewPoint software (Arrington Research). Due to technical difficulties of recording eye movements in the experimental environment (extensive video capture noise, too long setup time) we were only able to perform systematic eye-recording analyses in 10 out of 12 subjects in Experiment 1 and in 5 out of 7 subjects in Experiment 2. All eye movement analyses were performed off-line using custom routines written in Matlab (MathWorks). In brief, eye position samples were filtered using a second-order 10 Hz digital low-pass filter. Saccades were detected using an absolute velocity threshold (20 degrees per second), and blinks were defined as gaps in the eye position records caused by eyelid closure. Time periods with blinks were excluded from subsequent analysis. We instructed the subjects to continuously fixate on the central fixation spot. While our subjects fulfilled this requirement in the majority of trials, we still assessed the frequency of residual saccades (amplitudes ≥ 1 deg visual angle) on a trial by trial basis and compared saccade frequencies in the CUE and in the DELAY epoch across conditions to control for potential eye movement-related confounds.

### Experiment 1

The detailed paradigms of Experiment 1 are depicted in Fig. 1B. Each trial started with a 15 s or 16 s fixation period (FIXATE), during which subjects were instructed to fixate a centrally positioned fixation cross. In addition, subjects were required to perform “finger fixation” by placing their right index finger on a tactile cue on the touchscreen. This tactile cue also defined the starting position for reaching and it would corresponded to a location at the topmost position between the circles marking the reaching space. Eye blinks were allowed though discouraged during this period. Next, a CUE screen appeared for 1.5 seconds, indicating the experimental condition (a red central cue indicating CT and a green cue indicating DRT), a target location, reach direction (an arrow indicating clockwise or counterclockwise direction), eye and finger fixation points and instructed reach space boundaries (compare Fig. 1B). Both the starting location and all targets were positioned at a constant radius of about 3 deg visual angle from the fixation point. We used a predefined set of four target locations, placed in the upper-portion of the reach space either at −60° (=10 o’clock), −40°, +40° or +60°. Note that the starting position corresponds to 0° (=12 o’clock). This way, by manipulating reach direction and target location, we could alter the movement trajectory, without affecting target eccentricity and, accordingly, the hand-target difference vector. In the DRT condition subjects were required to remember the target location and to plan a movement to it according to the arrow cue. Subjects were told to ignore target and arrow cues in the CT condition, as the relevant cues would be delivered only later in the REACH phase. In both conditions subjects were asked to maintain fixation and avoid blinking during this CUE period. Next, we presented an image for 500 ms, which was made up of 400 randomly positioned, black and white circles approximately the size of the cursor, namely to mask any after-images of the cues (not shown in Fig. 1B). This mask was followed by a DELAY period lasting 15 s or 16 s. During the DELAY, subjects were instructed to keep fixation and, again, blinking was allowed though discouraged during that period. Note that we assume that correlates of goal-directed movement planning should be present during this phase in DRT but not in CT^19,20^. Finally, the response screen appeared for 3 s signaling the REACH phase. In the DRT subjects had to move their right index finger to the pre-cued target location as fast and as accurately as possible and through a single, smooth movement of their finger. In the CT subjects were presented a new set of target and arrow cues, and had to immediately perform a movement according to these two cues. Once reaching the instructed goal, subjects had to stay at the final location until the response screen disappeared. Then, a blank screen appeared for 4 s (not shown in Fig. 1B) and subjects had to return their finger to the tactile cue, i.e. the starting position. They were also encouraged to blink specifically during this period to reduce corneal drying in face of the prolonged periods of fixation in the preceding trial epochs. Note that visual feedback about finger position was only provided during the CUE and the REACH phase of a trial. All experimental conditions were presented randomly interleaved and were repeated 20 times across 5 consecutive scanning sessions per subject.

### Experiment 2

The overall design of Experiment 2 was similar to Experiment 1 (compare Fig. 1D). Each trial started with a 15–16 seconds fixation epoch. The (perceived) fixation for both eye and finger overlapped spatially and now corresponded to the center of the display. Then, a CUE screen was displayed for 1.5 s, with a task cue presented centrally at the fixation point (a red cue indicating CT and a green cue indicating DRT), and with a target cue in the periphery at about 3.2 deg or 7.2 deg visual angle distance for NEAR and FAR conditions, respectively. Target size in NEAR conditions was 0.8 deg visual angle. To accommodate for an increase in movement difficulty (ID) with increasing distance (D), we increased the size of the target (W) in the FAR conditions according to Shannon’s formulation of Fitts’ Law^49^, expressed as:

$${\rm{ID}}=\,\mathrm{log}\,2(D/{\rm{W}}+1)$$

In DRT trials, subjects were instructed to remember the peripheral target cue and to plan a movement to it, whereas in CT trials they were told to ignore the initial target cue. The CUE screen was then masked for 500 ms (compare Experiment 1 for details) and a DELAY period followed, lasting 15–16 seconds. Ultimately, the REACH screen appeared for 3 s and subjects had to move the cursor to the remembered target location in DRT, or to a newly cued target location in CT. After the instructed target location was reached, subjects had to maintain their finger position at this location until the end of this task period. Then the screen was blanked and subjects had to return their finger to the starting position (i.e. the initial fixation point for the finger, as was also defined by a tactile cue). Subjects were required to perform straight movements, without lifting the finger off the touchscreen and they were told to be “as fast and as accurate as possible”. Else they did not receive any additional instructions on how to plan/perform their reaches, as we did not want to bias their natural planning strategies. As in Experiment 1, we presented all experimental conditions randomly interleaved and repeated them 20 times across 5 consecutive scanning sessions per subject.

### Finger movement analysis

Finger movement data were preprocessed using custom routines programmed in Matlab (MathWorks) and analyzed statistically using R (R Foundation for Statistical Computing). In brief, during preprocessing we applied a digital low-pass filter (1^st^-order Butterworth filter; 6 Hz cut-off frequency). Data were analyzed to provide estimates of reaction times, movement accuracies, maximal velocities and movement durations. Reaction time was operationalized as the temporal difference between the onset of the movement epoch and the moment when finger velocity exceeded a threshold of 11 mm/s. Movement error sizes were characterized as the linear distance between the finger endpoint (calculated as average of the last five samples of the finger position during the REACH phase) and the nearest border of the target circle.

### fMRI acquisition and analyses

MRI images were acquired using a 3T Siemens TRIO scanner using a twelve-channel head coil (Siemens, Ellwangen, Germany). For each subject, we obtained a T1-weighted magnetization-prepared rapid-acquisition gradient echo (MPRAGE) anatomical scan of the whole brain (176 slices, slice thickness: 1 mm, gap: 0 mm, in-plane voxel size: 1 × 1 mm, repetition time: 2300 ms, echo time: 2.92 ms, field of view: 256 × 256, resolution: 256 × 256) as well as T2*-weighted gradient-echo planar imaging scans (EPI): slice thickness: 3.2 mm + 0.8 mm gap; in-plane voxel size: 3 × 3 mm; repetition time: 2000 ms; echo time: 30 ms; flip angle: 90°; field of view: 192 × 192 mm; resolution: 64 × 64 voxels; 32 axial slices. Overall, we obtained 2050 EPIs per subject in Experiment 1, which were collected during five consecutive runs. In Experiment 2 we collected again 2050 EPIs per subject over five runs. A single EPI volume completely covered the cerebral cortex as well as subcortical structures, apart from the most inferior aspects of the cerebellum which were not covered in several of our subjects. Functional data were processed using SPM8 (Wellcome Department of Cognitive Neurology, London, UK). In every subject, functional images were spatially aligned to the first volume in a series, and then coregistered to the T1 image. After that, a non-linear normalization of the structural image to a template in MNI space was performed. Parameters from normalization were then applied to the functional images. In the last step of data pre-processing, we smoothened all the functional images with a Gaussian filter of 6 mm × 6 mm × 8 mm FWHM.

In subject-specific fMRI analyses we next specified a GLM for each individual including our four experimental conditions (“task” [DRT, CT] x “movement distance” [“NEAR”, “FAR”]). Each condition was modeled separately for each of our three trial epochs (CUE + MASK, DELAY, REACH). The regressor duration was defined according to the respective epoch duration. The regressors were convolved with the canonical HRF-function of SPM8. Head motion parameters were included in the model as separate regressors. Fixation epochs weren’t explicitly modeled and served as an implicit baseline. The same principle procedure was applied both in Experiment 1 and in Experiment 2. To consider each subject’s individual functional brain organization, we detected planning areas significantly more active during the delay epoch in DRT than the respective epoch of CT trials in each subject (for that step, single subject activity maps were thresholded at p < 0.001, uncorrected). Individual ROI selection was guided by a group-level analysis, which we performed to delineate the areas commonly activated by reach planning across subjects in each of our experiments. For this purpose we entered the respective (first level) contrast images in the second-level group analyses (one-tailed t test). In this analysis-step, we used a minimal cluster-size criterion (k > 10 voxels) and a statistical threshold of p < 0.001, uncorrected.

### Region of Interest Analyses

We used the results of the group-level analysis and anatomical landmarks (see below) to initially identify reach planning-related areas. Our ROI set consisted of two main areas: left dorsal premotor cortex (PMd) located at the posterior end of the superior frontal sulcus, anterior to the hand area of M1; the left posterior-medial portion of superior posterior lobule (SPL)^50^. The additional movement planning ROIs included were: the left anterior end of the intraparietal sulcus (aIPS); the left middle intraparietal sulcus (mIPS); and left supplementary motor area (SMA)^16^. For each of these ROIs and for each individual we next identified the coordinate of the voxel exhibiting the local maximum of the individual subject statistical contrast DRT > CT that was closest to the respective ROI group-coordinate for the same statistical contrast. In addition we anatomically identified the hand area of left primary motor cortex^47^ due to its potential engagement in reach planning^8^ as well as left primary visual cortex (V1). The latter area served as a control for any activity related to visual stimulation, also because we are not aware of any findings showing its specific engagement in reach planning or execution. To avoid biasing our ROI-selection in individual subjects across both Experiments, as they were planning different movement types in each (circular [Exp. 1] vs. straight [Exp. 2]), in those subjects that participated in both of our experiments, we used the ROI coordinates of Experiment 1 also for Experiment 2 (5 out of 7 subjects, compare Figure S1). Please note that our ROI definition meets the criteria described by Kriegeskorte *et al*.^51^ to avoid circularity in data analysis. For ROI analyses we always considered the average activity of voxels within a 3 mm radius around the ROI center coordinate. We decided for univariate signal analyses, as it was sensitive enough to capture signal changes scaling up with trajectory parameters in our ROIs. We consider this to be the most direct and reliable way to make inferences about trajectory plan representations.

### Time-resolved fMRI analysis

Using custom protocols written in Matlab (MathWorks) (compare^20^), we extracted and analyzed BOLD-signal timecourses for each of our ROIs. Importantly, we separately analyzed timecourses during the CUE and DELAY vs. the REACH epoch, as the length of the delay period was temporally jittered. Timecourses for the CUE and DELAY phase were aligned to the onset of the CUE, and normalized to the baseline defined as a time window of −5 s to −3 s preceding CUE onset. As planning processes are likely to take place beginning as early as the presentation of the target cue we analyzed both trial epochs. The signals for the REACH epoch were aligned to the onset of the REACH phase and normalized to the same baseline period as was mentioned above. The timecourses were filtered with a digital high-pass filter (128 s cutoff value) and interpolated at 1 s temporal resolution (corresponding to the temporal jitter in our design).

To examine the effect of trajectory on the BOLD signal in each of the ROIs, we performed a time-resolved analysis of the timecourses with respect to their relative amplitude over the principal trial epochs (CUE/DELAY and REACH) using paired t-tests (compare “RESULTS” section). Only the significant differences spanning over three or more consecutive time points were taken into consideration and we limited the interpretation of the main results only to the late-delay phase of trials (see “RESULTS”) as the most relevant. The additional comparison between the main ROIs was done with mixed-models ANOVA in R. In order to additionally control for type II error, which is usually harder to control for with conventional statistics like ANOVA, we additionally calculated Bayes factors for our main and for our additional movement planning ROIs, using the method described by Dienes^52^. These factors allowed for comparing the hypotheses that planning signals are the same vs. different during late delay with respect to the trajectory conditions (NEAR vs. FAR) in each experiment. In particular, we assumed the signal differences to be approaching 0, when there is no difference between trajectory representations (null hypothesis), and diverging from 0 if the trajectory signals are different (alternative hypothesis). Specifically, for every ROI tested, we determined the Bayes factor based on subjects’ signal differences between FAR and NEAR conditions during the late delay period in each experiment. The alternative hypothesis was modeled by a uniform distribution (>0 to maximal signal difference observed within individual subjects for a given ROI). Bayes factors above 10 were considered as strong support for the hypothesis that trajectory-related planning signals differ, while values below 0.33 indicate strong support for the null hypothesis (no trajectory-related signal differences)^28^.

## Supplementary information

Supplement 1
