# Supplementary material for: Distinct contributions of human posterior parietal and dorsal premotor cortex to reach trajectory planning
Source: Sci Rep. 2019 Feb 13;9:1962. doi: 10.1038/s41598-019-39188-0 (PMC6374387; doi:10.1038/s41598-019-39188-0)
Supplement: Supplementary file 1 — Supplement 1 [file 41598_2019_39188_MOESM1_ESM.pdf]

# Distinct contributions of human posterior parietal and dorsal premotor cortex to reach trajectory planning

**Artur Pilacinski, Axel Lindner**

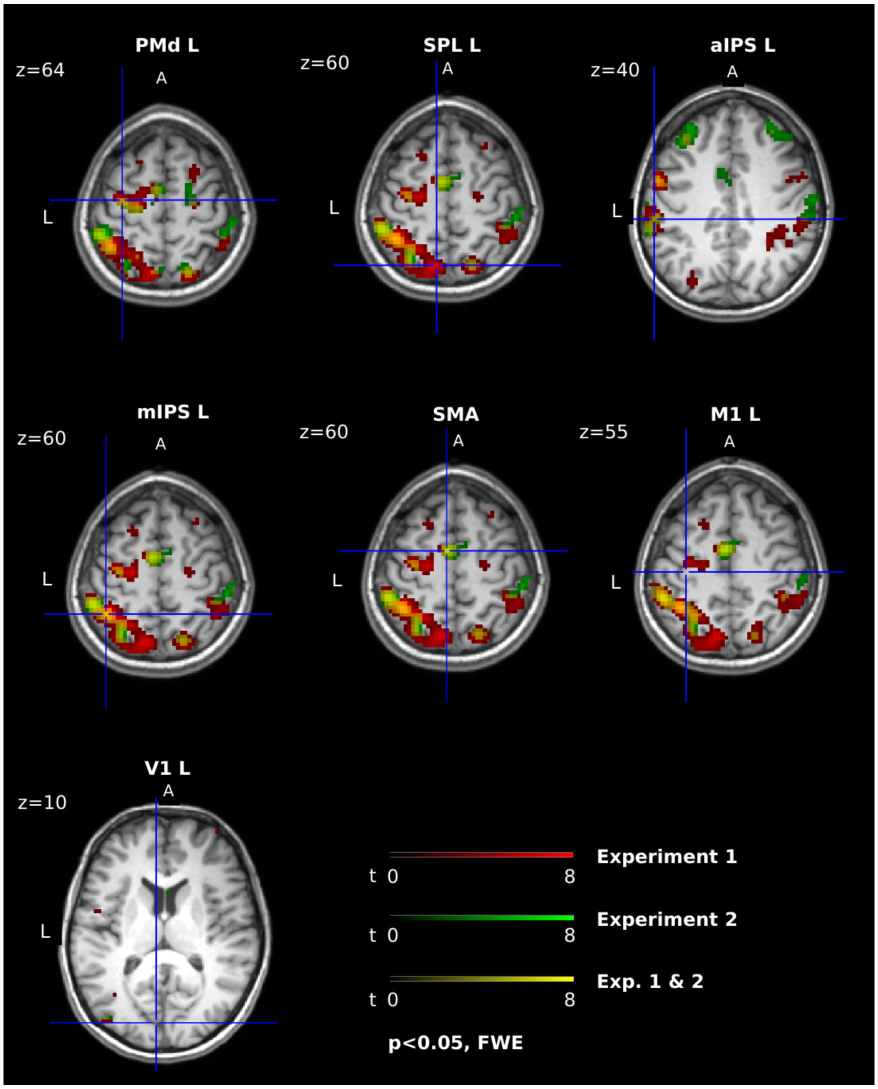


**Supplementary Figure S1.** Comparison of planning regions recruited by our two experiments in a representative subject (DRT>CT; the overlaid maps of activity were thresholded at $p<0.05$, FWE-corrected for multiple comparisons). Red and green shaded regions denote clusters of planning activity specific to the delay phase of Experiments 1 and 2, respectively. Yellow shaded regions represents areas active in both experiments. Blue crosshairs indicate centers of clusters selected for subsequent ROI analyses (compare METHODS).

**Supplement 1**

The qualitative assessment of the signal patterns in our additional ROIs suggested at first, that the SMA and aIPS encode the movement plans in similar way as SPL does. To test this, we performed an additional analysis of variance (factors: "EXPERIMENT", "DISTANCE" and "ROI") comparing PMd activity separately against SMA, against aIPS and against mIPS. The three-way interaction effect was present only in SMA activity (df=17, F=9.25, p=0.007, $eta^2_G$=0.02) but not in any of the intraparietal ROIs (mIPS: df=17, F=2.24, p=0.15, $eta^2_G$=0.003; aIPS: df=17, F=2.42, p=0.14, $eta^2_G$=0.009). Those, in turn showed a significant main effect of distance (mIPS: df=17, F=9.34, p=0.007, $eta^2_G$=0.01; aIPS: df: 17, F=10.45, p=0.005, $eta^2_G$=0.12).

In the next step, similar to comparing activity across experiments between PMd and SPL, we additionally calculated Bayes factors for SMA, aIPS and mIPS to assess presence of trajectory representations in these ROIs across experiments. For Experiment 1, the Bayes factors were: SMA: 0.4; aIPS: 0.34; mIPS: 0.34. This showed no evidence for engagement of any of these areas in complex trajectories planning. Bayes factors for Experiment 2 were: SMA: 32.13; aIPS: 65.62; mIPS: 4.10. The results for SMA were in agreement with both the t-tests and the ANOVA analysis and showed strong evidence for engagement of SMA in planning of straight but not curved trajectories. The apparent representation of straight trajectories in aIPS was confirmed by the Bayesian analysis, too. Apparently, however, the fact that the additional ANOVAs in both IPS regions did not reveal substantially differential trajectory signals across experiments, as were present in SPL and SMA, suggests that IPS activity differences in representing different trajectory plans were less pronounced than in SPL. We suspect that this may have likely resulted from the specific setting of our reaching task, relying on flexion of fingers and wrist muscles critically involved in prehension, and other precise hand movements. On the basis of other lines of research, we may speculate that the actual role for these IPS subregions was to represent the oncoming hand movement in terms of general finger control-related processes rather than in terms of the actual trajectory [1-4].

**Supplementary References**

1. Culham, J. C., Cavina-Pratesi, C. & Singhal, A. The role of parietal cortex in visuomotor control: What have we learned from neuroimaging? Neuropsychologia 44, 2668–2684 (2006).
2. Schaffelhofer, S., Agudelo-Toro, A. & Scherberger, H. Decoding a wide range of hand configurations from macaque motor, premotor, and parietal cortices. J. Neurosci. 35, 1068–81 (2015).
3. Jeannerod, M., Arbib, M. A., Rizzolatti, G. & Sakata, H. Grasping objects: the cortical mechanisms of visuomotor transformation. Trends in Neurosciences 18, 314–320 (1995).
4. Fogassi, L. & Luppino, G. Motor functions of the parietal lobe. Curr. Opin. Neurobiol. 15, 626–31 (2005).
